# New Therapeutic Approaches for the Treatment of Patients with Metabolic Dysfunction-Associated Steatotic Liver Disease (MASLD) and Increased Cardiovascular Risk

**DOI:** 10.3390/diagnostics14020229

**Published:** 2024-01-22

**Authors:** Marija Branković, Marija Dukić, Tijana Gmizić, Višeslav Popadić, Novica Nikolić, Ana Sekulić, Milica Brajković, Jelena Đokić, Edvin Mahmutović, Ratko Lasica, Marko Vojnović, Tamara Milovanović

**Affiliations:** 1 University Hospital Medical Center Bežanijska Kosa, 11000 Belgrade, Serbia; mdukic107@gmail.com (M.D.); tijana93@hotmail.com (T.G.); viseslavpopadic@gmail.com (V.P.); novica.nikolic87@yahoo.com (N.N.); sekulic.ana@bkosa.edu.rs (A.S.); brajkovic.milica@yahoo.com (M.B.); drjelenadjokic@gmail.com (J.Đ.); 2 Faculty of Medicine, University of Belgrade, 11000 Belgrade, Serbia; drlasica@gmail.com (R.L.); tamara.alempijevic@med.bg.ac.rs (T.M.); 3 Department of Internal Medicine, General Hospital Novi Pazar, 36300 Novi Pazar, Serbia; edvin.mahmutovic@outlook.com; 4 Department of Cardiology, Emergency Center, University Clinical Center of Serbia, 11000 Belgrade, Serbia; 5 Clinic of Gastroenterology and Hepatology, University Clinical Center of Serbia, 11000 Belgrade, Serbia; marko.vojna@gmail.com

**Keywords:** MASLD, cardiovascular diseases, metabolic syndrome, pharmacotherapy

## Abstract

Metabolic dysfunction-associated steatotic liver disease (MASLD) was previously known as nonalcoholic fatty liver disease (NAFLD). The main characteristic of the disease is the process of long-term liver inflammation, which leads to hepatocyte damage followed by liver fibrosis and eventually cirrhosis. Additionally, these patients are at a greater risk for developing cardiovascular diseases (CVD). They have several pathophysiological mechanisms in common, primarily lipid metabolism disorders and lipotoxicity. Lipotoxicity is a factor that leads to the occurrence of heart disease and the occurrence and progression of atherosclerosis. Atherosclerosis, as a multifactorial disease, is one of the predominant risk factors for the development of ischemic heart disease. Therefore, CVD are one of the most significant carriers of mortality in patients with metabolic syndrome. So far, no pharmacotherapy has been established for the treatment of MASLD, but patients are advised to reduce their body weight and change their lifestyle. In recent years, several trials of different drugs, whose basic therapeutic indications include other diseases, have been conducted. Because it has been concluded that they can have beneficial effects in the treatment of these conditions as well, in this paper, the most significant results of these studies will be presented.

## 1. Introduction

This is a review paper in which we try to show the connection between metabolic dysfunction-associated steatotic liver disease (MASLD) and cardiovascular diseases (CVD) based on the current knowledge. The Medline and PubMed databases were searched. Papers were searched according to the keywords, “MAFLD”, “NAFLD”, “NASH”, “fatty liver disease”, “cardiovascular diseases”, “metabolic syndrome”, “pharmacotherapy”, and “cirrhosis”. Both review and original articles were taken into account.

MASLD is a disease that occurs with an increasing frequency in Western countries [1]. Because of its consequences, it is becoming an increasingly common reason for liver transplantation [2]. The basis of MASLD is the accumulation of lipids in the hepatocytes, which is not a consequence of alcohol abuse, the effects of drugs, or other agents [3]. Several studies have shown that there is a connection between metabolic diseases, such as diabetes mellitus, insulin resistance, and obesity, but also cardiovascular disorders [1,3], which is why nonalcoholic fatty liver disease (NAFLD) is now known as MASLD. Rinella et al., through a multisociety Delphi consensus statement on new fatty liver disease nomenclature, published various articles about replacing the term, “nonalcoholic fatty liver disease”, with the term, “metabolic-associated steatotic liver disease”. They explained the proposed change by saying that this enables a clearer connection between metabolic syndrome and fatty liver disease, given that we know that metabolic syndrome is the most important etiological factor for the development of NAFLD [4]. The first criteria for diagnosing MASLD were established in 2020 [5]. The diagnosis of MASLD, according to these criteria, implies hepatic steatosis, which is confirmed with imaging methods, histology, or biomarkers with the presence of obesity, diabetes, and at least two of the following seven criteria: increased waist circumference (>102–88 for Caucasian men and women, >90/80 for Asian men and women), arterial hypertension (>130/85 mmHg or drug treatment), hypertriglyceridemia (>150 mg/dL or specific drug treatment), low HDL cholesterol (<40 mg/dL for men and <50 mg/dL for women), prediabetes (HbA1c 5.7–6.3 or fasting blood glucose 100–125 g/dL), insulin resistance (HOMA > 2.5), or high sensitivity C reactive protein (2 mg/L). The advantages of the MASLD criteria over NAFLD are that MASLD correlates better with the risk of liver fibrosis, hepatocellular carcinoma, and extrahepatic malignancy [6,7]. As for the pediatric population, given that children do not consume alcohol, the term, NAFLD, is certainly less appropriate than MASLD. The histological differences between MASLD in pediatric and adult populations are also evident. In adults, the inflammation is usually lobular in localization, while in children it is portal. The ballooning of hepatocytes is common in adults and rare in children. Fibrosis occurs perisinusoidally in adults, while in children, it is portal and periportal localized [8]. It is believed that these differences originate from genetic factors that may be different for the adult and pediatric forms. The prevalence of MASLD in adults is higher than in children (24% vs. 7.6% according to some studies). 

As we have already mentioned, these conditions are more often in elderly patients, in which liver diseases occur with certain specificities [9]. In addition, patients who have MASLD or metabolic dysfunction-associated steatohepatitis (MASH), particularly in cases of excessive alcohol use, have a higher risk of developing alcohol-associated liver disease (ALD) and its complications [5]. Common to metabolic diseases, cardiovascular diseases, MASLD, and MASH have a disorder in lipid metabolism [3,6,7]. MASLD is associated with an increased influx of free fatty acids (FFA) into the liver, an accumulation of FFA in the form of lipid droplets, and a stimulation of de novo lipogenesis. The result is changes in the lipid profile, primarily in terms of an increase in the synthesis of very low-density lipoproteins (VLDL), a decrease in the level of high-density lipoproteins (HDL), and a predominance of low-density lipoproteins (LDL). This change in the lipid profile favors harmful consequences, such as the development of cardiovascular complications [3]. Changes in the lipid profile in pediatric MASLD are primarily genetically determined, and there are several genetic forms of MASLD, depending on the dominant type of genetic alteration that is present [5].

The accumulation of lipids, more commonly triglycerides, in the cytoplasm of hepatocytes leads to the damage of the organelles and finally apoptosis of hepatocytes. This briefly illustrates the lipotoxic effect that, along with oxidative stress, results in chronic inflammation in the liver that is responsible for the progression of MASLD to MASH [3,6]. The difference between these two diseases is the existence of inflammation and fibrosis, in which the last stage of liver damage is cirrhosis [8]. Patients with MASH are at an increased risk of hepatocellular carcinoma [9]. According to the previous classification, the progression of MASLD leads to the emergence of MASH, which represents, in other words, its inflammatory form. In about 20% of cases, MASH progresses to liver cirrhosis. It is important to say that patients with confirmed MASH are at a higher risk of developing hepatocellular carcinoma compared to those with MASLD. The occurrence of hepatocellular carcinoma, in this case, does not have to be exclusively related to the stage of cirrhosis, that is, the progression of MASH to cirrhosis. The reason for this may be that MASH, i.e., MASLD, has multifactorial genesis, and different factors have an impact on this progression. Chronic inflammation is the main reason for the transformation of hepatocytes and the beginning of the process of carcinogenesis. MASH-related hepatocellular carcinoma occurs in approximately 2% of cases per year and differs from other forms of this tumor in terms of its molecular and immunological characteristics. According to some studies, it occurs with the same frequency in people of both sexes [10].

Also, as a result of chronic inflammation, liver parenchymal cells are damaged, resulting in a reparation process, which, in this case, represents fibrosis. Stellate, Kupffer, and endothelial cells participate in the process of fibrogenesis. Macrophages and dendritic cells also contribute to the process. The role in matrix production belongs to stellate cells, which are activated into myofibroblasts thanks to mediators that arise due to the manifestation of the lipotoxic effect of lipids at the level of the liver. Over time, and under the influence of many factors, the fibrogenesis process progresses, causing liver fibrosis and, as the final stage, cirrhosis [11]. In recent years, there has been an increasing number of studies that deal with finding ways to inhibit stellate cells, that is, to stop the process of liver fibrosis. The autophagy of stellate cells was singled out as a new important process. Research has shown that stellate cells release vesicles containing fibrogenic proteins. By inhibiting the release of these vesicles, the process of fibrogenesis would be slowed down [12]. Previous research on this topic has shown promising results. It is also interesting that the content of these vesicles has also been associated with the progression of hepatocellular carcinoma [13].

**Figure 1:**
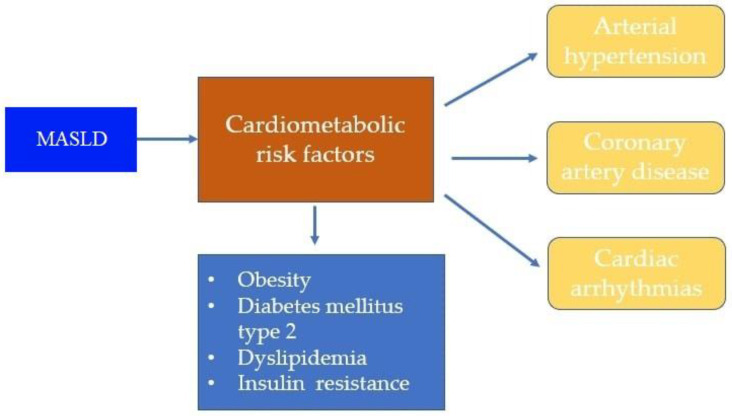
Metabolic dysfunction-associated steatotic liver disease (MASLD) and cardiometabolic risk factors.

Despite the prevalence and possibility of liver cirrhosis and all these complications, there is still no approved pharmacotherapy for MASLD and MASH. The basis of the association between MASLD/MASH and CVD is complex and rests on common pathophysiological mechanisms and risk factors [14,15] (Figure 1). 

Metabolic syndrome combines insulin resistance, diabetes mellitus type 2 (T2DM), obesity, and hypertriglyceridemia, and steatotic liver disease is considered the hepatic manifestation of metabolic syndrome [16]. As part of metabolic syndrome, fat accumulation occurs not only in the liver, but also perivascularly, peripancreatically, in the pericardium and epicardium, and around the kidneys, skeletal muscles, and other organs [17]. An increase in the amount of visceral fat deposits leads to an increase in cardiovascular risk [16]. The process of lipotoxicity is not only related to the accumulation of lipids in the liver. This process is also found at the level of the myocardium, as it has been observed, and more often in obese people and diabetics [18]. In a situation where there is less consumption of free fatty acids by cardiomyocytes compared to their uptake from the blood, fatty acids accumulate in the myocardium [19]. Free fatty acids are metabolized in cardiomyocytes primarily by oxidation in the mitochondria. However, in the case where the flow of free fatty acids is increased, the mitochondria are damaged, and various signaling pathways are activated, initiating the process of lipotoxicity [20,21]. In conditions where there is an increased accumulation of lipids in cardiomyocytes, the capacity of the mitochondria for their metabolism is exceeded. The capacity of the cells for their storage is also surpassed. Mitochondria, in addition, are a source of reactive oxygen species (ROS), so in conditions where there is an increased accumulation of free fatty acids, the risk of lipid peroxidation is increased. Lipid peroxidation creates free radicals that damage the DNA molecule, proteins, and lipids of the mitochondrial and cell membranes [22]. This is the basis for the lipotoxicity process at the cardiomyocyte level. As previously stated, consequently, MASLD progresses to MASH in a long-term process of inflammation and consequent fibrosis. This occurs in 10–25% of patients with MASLD, further causing a variety of complications that can significantly affect morbidity and mortality [23].

## 2. Association between Metabolic Dysfunction-Associated Steatotic Liver Disease (MASLD)/Metabolic Dysfunction-Associated Steatohepatitis (MASH) and Cardiovascular Diseases (CVD)

Atherosclerosis is a multifactorial disease that is characterized by endothelial dysfunction, thinning of the arterial walls, and an increase in the risk of cardiovascular and cerebrovascular diseases, primarily myocardial infarction and cerebrovascular infarction [24]. It has been proven in several studies that with the progression of MASLD into MASH, there is also a pronounced progression of atherosclerosis [25]. MASH is a significant risk factor for the development of carotid atherosclerosis, endothelial dysfunction, arterial stiffness, coronary calcification, and impaired left ventricular function [26,27]. Endothelial dysfunction involves the activation of endothelial cells, which, in their activated form, produce cytokines that lead to the transition of monocytes into macrophages at the level of the intima media. They use their receptors to bind and then modify the low-density lipoprotein molecule (LDL) and release chemokines and proinflammatory cytokines [28]. This allows the inflammatory process to spread to other tissues and organs. The consequence of vascular inflammation and oxidative stress is a decrease in the level of nitric oxide (NO), which has a vasodilatory role, and a subsequent increase in the level of its antagonist, asymmetric dimethyl arginine (ADMA) [29,30]. In this way, a disturbance of vasomotor regulation and vascular permeability occurs [30]. Elevated levels of ADMA are found in patients with MASLD [26]. Patients with MASLD have a higher risk of developing atherosclerosis, primarily coronary artery disease [31]. MASLD/MASH is often associated with one or more factors of metabolic syndrome (hyperinsulinemia, consequent hypertriglyceridemia, T2DM, obesity), and CVD are the main cause of mortality in these patients [32]. Apart from the accumulation of lipids in the liver and peripancreatic region (which leads to insulin resistance and the onset of T2DM), the accumulation of lipids in the epicardium stood out as significant. Epicardial adipose tissue (EAT) is a special accumulation of lipids located between the visceral layer of the epicardium and the myocardium. At birth, it has the characteristics of brown adipose tissue and acts protectively. However, in pathological conditions, such as MASLD/MASH, coronary artery disease, atrial fibrillation, and heart failure, it becomes extremely secretory and active. Its products have a paracrine and vasocrine effect. Most of them are proinflammatory and profibrotic cytokines that have a proatherogenic and proarrhythmogenic effect. Insight into the importance of EAT contributed to the development of new therapeutic options for the treatment of the mentioned CVD by modifying the previously known EAT factors [33].

## 3. Common Cardiovascular Diseases (CVD) in Patients with Metabolic Dysfunction-Associated Steatotic Liver Disease (MASLD)

### 3.1. Arterial Hypertension

Studies have shown that there is an association between arterial hypertension and MASLD [34,35]. Aneni et al. conducted a cohort study of 5362 middle-aged women and men in Brazil with the aim of determining the association between hypertension and MASLD [36]. Patients were divided into three groups based on blood pressure values: a group with normal blood pressure, a group with prehypertension, and a group of hypertensive patients. Among the included patients, MASLD was present in 36.2%. The prevalence of MASLD was highest among hypertensive patients. Patients with hypertension were more likely to have a higher Fibrosis-4 score compared to those with prehypertension and normal blood pressure values. It has been shown that MASLD can be an independent risk factor for the development of hypertension, independent from the effects of other metabolic factors. Ryoo et al., unlike the aforementioned study, compared blood pressure values with the degree of liver steatosis. It was found that the occurrence of hypertension is more frequent and more certain with an increase in the degree of steatosis, i.e., severity of MASLD [37]. Another study examined the relationship between MASLD and hypertension. Latea et al. followed 35 patients for four years. The patients were divided into four groups depending on their blood pressure values. Serum insulin values were monitored in all groups. It was concluded that more pronounced insulin resistance occurs in those with more pronounced changes in blood pressure and that this may be the connection between hypertension and MASLD [38].

### 3.2. Coronary Artery Disease

Considering the connection between MASLD and atherosclerosis, the conclusion regarding the connection between MASLD and coronary artery disease is imposed. Patients with MASLD have an increased frequency of clinically manifested atherosclerosis, which most often manifests as a myocardial infarction or symptoms of ischemic heart disease. One of the larger meta-analyses was conducted by Mahfood Haddad et al. and has been repeatedly cited in current scientific works. It included 25,837 patients and showed that patients with MASLD were at a greater risk for coronary artery disease compared to those without MASLD [31]. Ren et al. conducted an interesting study in which they assessed the association between genetically determined MASLD and the risk of coronary artery disease [39]. Their results indicated an almost certain connection between these two diseases. On the other hand, Peng et al. came up with different results. Their study did not prove a causal relationship between coronary artery disease, heart failure, and stroke [40]. Other studies have shown that patients with MASLD are at a greater risk for the development of unstable atherosclerotic plaque, its complications, and the occurrence of an acute coronary event [41,42].

### 3.3. Cardiac Arrhythmias

Several studies have shown that MASLD brings an increased risk of cardiac arrhythmias. Atrial fibrillation occurs most often, both persistent and paroxysmal, and it can significantly affect the mortality of these patients [43,44,45,46,47]. In addition to atrial fibrillation, ventricular rhythm disorders are also more common, given that changes in the secretory activity of pericardial and epicardial adipose tissue can lead to changes in the function of ion channels [48]. For this reason, QTc prolongation occurs more often in patients with MASLD [49]. A study conducted in Taiwan that included about 30,000 patients with MASLD showed an association between MASLD and an increased risk of QTc prolongation [50]. Mantovani et al. showed that MASLD is an independent risk factor for the occurrence of ventricular arrhythmias and premature ventricular complexes [51].

### 3.4. Heart Failure

The relationship between MASLD and heart failure (HF) is still unclear, but some studies have shown that patients with MASLD are at a greater risk for developing HF. One such study is a retrospective study conducted by Fudim et al. [52]. This study included Medicare beneficiaries who had one inpatient or two outpatient claims using the International Classification of Diseases, Ninth Revision, Clinical Modification (ICD-9-CM) claim codes in support of MASLD. An exclusion criterion was previously used for HF. Patients were monitored from October 2015–December 2016, and the diagnosis of HF was made in those who had at least one inpatient or at least two outpatient HF claims during that period. According to this study, patients with MASLD had a higher risk of developing HF, more often HF with a preserved, rather than a reduced, ejection fraction. A meta-analysis by Mantovani et al. aimed to quantify the risk of HF in patients with MASLD [53]. They came to the conclusion that MASLD is associated with a moderately higher risk of HF, independent of age, gender, ethnicity, presence of hypertension, diabetes, and other cardiovascular risk factors. More precisely, patients with MASLD have a 1.5 times higher long-term risk of developing HF.

## 4. New Therapeutic Approaches for the Treatment of Metabolic Dysfunction-Associated Steatotic Liver Disease (MASLD)/Metabolic Dysfunction-Associated Steatohepatitis (MASH) and Their Effects on Cardiovascular Risk Reduction

The treatment of patients with MASLD/MASH involves changing life habits, diet, and weight loss [1,54]. Regardless, a large number of studies are being conducted with the goal of finding an effective pharmacotherapy that would prevent the progression of MASLD to MASH, and then the occurrence of liver cirrhosis. The treatment of these conditions has a positive effect on the cardiovascular system and the reduction of cardiovascular risk. The main goal of adequate pharmacotherapy should be the inhibition of lipid accumulation in hepatocytes, cardiomyocytes, and other cells, but also anti-inflammatory and antifibrotic effects. Some of the potential solutions are farnesoid X receptor (FXR) agonists, inhibitors of de novo fat synthesis, peroxisome proliferator-activated receptor agonists (PPAR), and analogues of fibroblasts growth factor (FGF) [54] (Table 1).

### 4.1. Farnesoid X Receptor (FXR) Agonists

**Figure 2:**
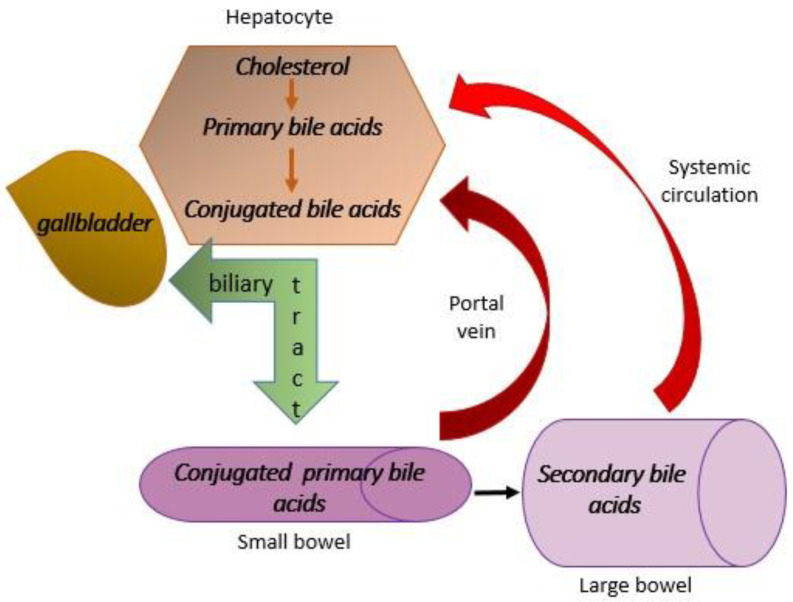
Enterohepatic circulation. Primary bile acids and cholic and deoxycholic acid are produced from cholesterol in the hepatocytes. Also, conjugation with glycine and taurine takes place there, and then the conjugated primary bile acids go to the gallbladder via the biliary tract, where they are deposited. Under the influence of cholecystokinin, which is released after a meal rich in fat, the conjugated primary bile acids go to the small intestine through the biliary tract. Over 90% is resorbed here and returns to the liver via the portal vein. About 1–2% flows into the large intestine where, under the influence of intestinal microbiota enzymes, it is transformed into secondary bile acids, which are transported back to the liver through systemic circulation.

The farnesoid X receptor (FXR) is a receptor that participates in the regulation of the synthesis and enterohepatic circulation of bile acids [55]. Bile acids are cholesterol derivatives consisting of cholic (CA) and chenodeoxycholic acid (CDCA). They are also called primary bile acids. By their nature, these acids are poorly soluble, and in the process of conjugation with glycine and taurine, they become conjugated. In that state, these acids are more soluble and can participate in enterohepatic circulation [56]. They are deposited in the gallbladder and released under the influence of cholecystokinin (CCK) after a fat-rich meal. Their main role is to emulsify fats from food for easier absorption [57]. The largest part of conjugated bile acids, more than 90%, is resorbed via the portal vein back to the liver, and only 1–2% passes into the large intestine and, thus, bypasses enterohepatic circulation [58,59]. In the colon, enzymes of the intestinal microbiota convert primary bile acids into secondary bile acids, namely deoxycholic acid (DCA), which is formed from CA, and ursodeoxycholic (UCA) and lithocholic acid (LCA), which are formed from CDCA. Secondary bile acids are more lipophilic than primary bile acids and, as such, are resorbed at the level of the colonic mucosa and reach the liver through systemic circulation [58,59] (Figure 2). FXR is localized in the liver and intestine. Its main role is the inhibition of the conversion of cholesterol into primary bile acids, that is, the inhibition of their synthesis from cholesterol. In addition, the activation of this receptor has a role in preventing the resorption of bile acids at the level of the ileum, which affects enterohepatic circulation. This is the basis of the modulation of the inflammatory process underlying MASH as well as the fibrosis process.

The most studied FXR agonist is obeticholic acid (OCA) which is approved for the treatment of primary biliary cirrhosis when ursodeoxycholic acid is not sufficient. It is a synthetic analogue of CDCA that activates FXR with greater potency [60]. In the same way as CDCA, OCA is conjugated and then undergoes enterohepatic circulation. By activating FXR, in addition to the mentioned decrease in the synthesis of primary bile acids and their reduced resorption from the ileum, a reduction in lipogenesis and gluconeogenesis, an anti-inflammatory and indirect antifibrotic effect, is achieved [61,62]. The anti-inflammatory effect is achieved by reducing the level of inflammatory mediators in the endothelial cells of the liver sinusoids and stellate cells, which also have an indirect antifibrotic effect. The two main studies that studied the effect of OCA in the treatment of MASH are the REGENERATE study and the FLINT study.

The REGENERATE study was a multicenter, randomized, double-blind, placebo-controlled study conducted in patients with a histopathology-verified diagnosis of MASH who have stage 2 or 3 fibrosis or stage 1 fibrosis with at least one of the accompanying comorbidities [63]. The effect of OCA and the placebo on histological findings, MASH activity, and clinical outcomes that are related to the liver were compared. Patients received a placebo, 10 mg, or 25 mg of OCA orally. A liver biopsy was performed at the time of screening, after 18 weeks, after 48 weeks, and at the end of the study. A total of 1968 patients with MASH were included in the study. Of these, 931 patients had stage 2 or 3 fibrosis. In the group that received a placebo, 12% of patients showed fibrosis improvement. In the group that received 10 mg of OCA, 18% of patients showed fibrosis improvement, and from those that received 25 mg of OCA, 23% of patients showed improvement. The ultimate goal of the resolution of MASH was not achieved in all patients, but it was concluded that treatment with 25 mg of OCA led to a significant improvement in fibrosis and a reduction in activity in patients with MASH. The most common side effect of therapy was pruritus.

The FLINT trial was a multicenter, double-blind, placebo-controlled, randomized clinical trial conducted in the United States of America. Patients who were diagnosed with biopsy-proven MASH were included, but those with liver cirrhosis were excluded [64]. This study compared the effects of an oral administration of OCA at a dose of 25 mg versus a placebo for 72 weeks. The conclusion was that OCA therapy led to a significant histological improvement in patients with MASH, but the degree of resolution of MASH was not statistically significantly different in patients who received a placebo compared to those who received OCA.

Another lesser-known FXR agonist is tropifexor (TXR). This drug belongs to the group of nonspecific FXR agonists and participates in the induction of the FXR gene. It has been shown to be a potent activator of these genes in vivo in rodent models. A multicenter, randomized, double-blind study named TANDEM was conducted to evaluate the efficacy of TXR and cenicriviroc as a monotherapy or combination therapy in patients with histopathology-verified MASH [65]. The conclusion was that the use of both drugs led to an improvement in fibrosis and a resolution of MASH, and that the combined therapy was more potent than monotherapy.

### 4.2. Peroxisome Proliferator-Activated Receptor (PPAR) Agonists

Peroxisome proliferator-activated receptors (PPAR) are receptors that participate in the regulation of lipid and glucose homeostasis and are located in the cell nucleus. Their activation leads to the modulation of metabolic pathways and, among other things, the reduction of gene expression for pro-inflammatory molecules. There are three types of receptors: PPARα, PPARβ/δ, and PPARγ [66]. 

PPARγ agonists are thiazolidinediones (TZDs) that are found in adipose tissue and promote the uptake of free fatty acids and their storage in the form of triglycerides [66]. PPARγ also regulates the release of adipokines from adipocytes [67]. It has been shown that the activation of PPARγ by TZDs reduces the degree of inflammation and fibrosis of the liver by keeping fatty acids in the periphery, which prevents their deposition in hepatocytes and, thus, the lipotoxic effect of lipids, which is fundamental in the pathogenesis of MASLD/MASH [68]. The main representative of this group is pioglitazone. The basic role of pioglitazone is the regulation of elevated glycemic values; however, it has been observed that it can have a positive effect in patients with histopathology-verified MASH. It led to the resolution of MASH and reduction in the stage of fibrosis, but adverse effects limited its application. Fluid retention occurred, which increases the risk of heart failure, weight gain, and the appearance of osteopenia in older women, multiplying the possibility of pathological fractures [68,69].

Saroglitazar is an agonist of α and γ PPAR. Its usual use is for the treatment of insulin resistance and dyslipidemia, but it has also been shown to have beneficial effects in the treatment of MASLD/MASH [70,71,72]. A prospective, multicenter, double-blind, placebo-controlled study was conducted on this topic. The study included 106 patients with MASLD/MASH who had a body mass index ≥25 kg/m^2^ and an alanine aminotransferase (ALT) value ≥50 U/L [73]. They were divided into three examined groups and one control group that received a placebo. The examined groups differed in the dose of the drug that the patients received, that is, 1 mg, 2 mg, or 4 mg of saroglitazar for 16 weeks. A significant improvement in ALT values, a decrease in liver fat content, which was monitored by magnetic resonance imaging (MRI), and a better regulation of dyslipidemia and insulin resistance were found. The use of this drug proved to be safe, and the patients tolerated it well [72].

Elafibranor is an agonist of PPARα and PPARδ receptors and belongs to the group of fibrates, drugs used in the treatment of elevated triglyceride levels. In addition, it significantly affects glucose homeostasis, so its use has found its place in the treatment of insulin resistance [74]. To date, several studies have been conducted investigating the impact of elafibranor in the treatment of MASLD/MASH. The GOLDEN study was a multicenter, double-blind, randomized, placebo-controlled study that compared the effects of a placebo with the oral administration of 80 mg and 120 mg of elafibranor [75]. The exclusion criterion in this study was the existence of liver cirrhosis. In patients who received elafibranor as a dose of 120 mg, there was a higher percentage of a resolution of MASH and a reduction in the stage of fibrosis. During administration, this drug caused an increase in serum creatinine, but this was reversible. The drug was generally well-tolerated. Other drugs from the fibrate group have been tested with the same goal, but so far, no significant observations have been made. Some of these drugs are gemfibrozil, fenofibrate, pemafibrate, and lanifibranor [74].

Drugs from the group of glucagon-like peptide (GLP)-1 agonists that are normally used in the treatment of type 2 diabetes mellitus, such as semaglutide, liraglutide, and tirzepatide, are also potentially useful drugs in the treatment of MASLD/MASH due to their numerous metabolic effects [70,74].

Semaglutide is a drug that, as previously mentioned, is approved for the treatment of type 2 diabetes mellitus and is particularly suitable due to its weekly regimen [76]. This drug reduces the cardiovascular risk in patients with diabetes, but also in cardiovascular patients. This is important considering that the most common cause of death in patients with metabolic diseases is of a cardiovascular origin [76]. So far, a large number of studies have been conducted after it was observed that semaglutide leads to significant weight loss in patients taking it, which also enables the aforementioned role. Semaglutide can be administered subcutaneously or orally, but it has been proven that the oral form has a less potent effect on weight loss compared to the subcutaneous administration [77]. The most common side effects of using this drug were nausea, vomiting, diarrhea, and constipation, but they occurred with the same frequency in patients in the tested group and group of patients who received a placebo and were transient. Between November 2016 and March 2020, a trial was conducted in several countries with the aim of evaluating the role of semaglutide in the treatment of patients with histopathology-verified MASH with fibrosis stage 1, 2, or 3. It was a prospective, multicenter, randomized, double-blind, placebo-controlled study that included a one-day subcutaneous administration of semaglutide at a dose of 0.1 mg, 0.2 mg, or 0.4 mg or the administration of a placebo for 72 weeks [78]. A higher percentage of patients that received semaglutide had a resolution of MASH compared to those that received a placebo: 40% of those who received a dose of 0.1 mg, 36% of those who received a dose of 0.2 mg, and 59% of patients who received a dose of 0.4 mg of semaglutide. However, no significant difference was observed in the degree of fibrosis before and after the applied therapy [78].

The LEAN study was conducted with the aim of investigating the effect of liraglutide in patients with clinical signs of MASH who are obese [79]. As stated in the study design, A’Hern’s single-group method was used, which required 38% (8/21) of successes in the liraglutide group for the effect of liraglutide to be considered clinically significant. Between August 2010 and May 2013, 26 patients received liraglutide at a dose of 8 mg, and 26 of them received a placebo for 48 weeks. The ultimate goal was a resolution of MASH without worsening the fibrosis. Approximately 39% of patients who received liraglutide had a proven resolution of MASH, but this percentage was lower in the control group, only 9% of patients. The most common side effects of liraglutide were from the gastrointestinal tract, i.e., diarrhea, constipation, and a loss of appetite. The impact of liraglutide was also evaluated on the pathohistological findings in patients with MASH. In some cases, histological resolution was demonstrated, but additional studies are needed with the aim of obtaining more precise data [80].

The third drug from the group of GLP-1 agonists is tirzepatide. This drug is also a GIP (glucose-dependent insulinotropic polypeptide) agonist. It has shown an effect on the reduction of steatosis and fibrosis in patients with MASLD/MASH, as well as an effect on weight loss, but additional research is necessary to determine the importance of this drug in the treatment of MASLD/MASH [81,82]. It is important to mention the role of GLP-1 agonists in weight loss, given that weight loss has a positive effect on glycoregulation and reducing the risk of complications. These drugs have a positive effect on weight loss by providing a longer feeling of satiety by slowing the passage of food from the stomach to the small intestine, reducing the release of glucagon after a meal, and reducing the need for food intake [81].

### 4.3. Analogues of Fibroblast Growth Factors (FGF)

Analogues of fibroblast growth factors (FGF) are an important group of drugs considering that they have important metabolic roles and regulate the metabolism of bile acids, glucose homeostasis, and energy turnover in the cell [70,74]. FGF are signal proteins that act through their receptors (FGF receptors or FGFR) which are in group of the tyrosine kinases. One of the most studied is FGF19, which is characterized by a reduced affinity for heparin sulfate. Aside from FGF19, others in this group are FGF21 and FGF23. In addition to autocrine and paracrine signaling, a reduced affinity for heparin sulfate, which is located intracellularly, enables it to act at a place far from the place of origin, i.e., endocrine action. It fulfills its endocrine role through FGFR4 and coreceptors α-Klotho or β-Klotho [83]. FGF19 can promote hepatocyte proliferation and inhibit bile acid synthesis [84]. In patients with MASLD/MASH, fats are deposited in the hepatocytes in the form of droplets, and they have a lipotoxic effect. Part of the lipotoxic effect is oxidative stress and the formation of free radicals that damage hepatocytes. This stimulates the production of FGF, such as FGF21, which then has a paracrine and autocrine effect on hepatocytes. It has been proven that the level of this hormone is increased by 10–20 times in patients with MASLD/MASH but also in patients suffering from cardiovascular and metabolic diseases. FGF21 participates in the maintenance of metabolic homeostasis, reduces body weight and the level of triglycerides in the liver and systemic circulation, regulates the influence of insulin on the level of glucose, depending on the needs, and so on [85]. Pegbelfermin and efruxifermin are synthetic analogues of FGF21 that have been investigated in patients with MASH in several clinical studies. Their effects were compared with the placebo. Both drugs have been shown to reduce liver fat content, but more detailed studies are underway [74,86].

Aldafermin is an engineered analogue of the gut hormone FGF19. In the ALPINE 2/3 study, which was designed as a randomized, double-blind, placebo-controlled phase 2b study, 171 patients with histopathology-verified MASH and stage 2 or 3 fibrosis were included. This drug has been shown to improve the stage of fibrosis by at least one grade after 24 weeks of treatment. Adverse effects were mild to moderate, and diarrhea was the most common [87]. This drug was investigated in another study with the same design and compared the subcutaneous administration of aldafermin with the administration of a placebo [88]. Patients had a biopsy-confirmed diagnosis of MASH. The study group received the drug in a dose of 3 mg or 6 mg, while patients from the control group received a placebo. The first goal was the reduction of liver fat content after up to 12 weeks of therapy, which was assessed by MRI. As many as 74% of patients who received the drug in a dose of 3 mg and 79% of patients who received the drug in a dose of 6 mg achieved a reduction in liver fat content of at least 5% during the 12-week therapy. After the analysis of the control biopsies of the liver, an improvement in histological findings was registered in terms of reduction of steatosis and fibrosis. Given the above, several studies were conducted that examined the role of the drug aldafermin in the treatment of metabolic and cholestatic liver diseases [88].

### 4.4. The Other Drugs Which Have Beneficial Effects

As we said at the beginning, the main goal of adequate pharmacotherapy, in addition to the inhibition of fat deposition in hepatocytes, is also the inhibition of de novo synthesis of lipids in the liver [70]. One of the drugs that has this role is arachidyl amido cholanoic acid (Aramchol). Two larger studies that examined its effect in patients with MASH are ARREST and ARMOR.

The ARREST trial was a randomized, double-blind, placebo-controlled phase 2b study in which 247 patients with MASH received Aramchol at a dose of 400 mg or 600 mg or a placebo for 52 weeks [89]. The first objective was the reduction of liver triglycerides after 52 weeks of therapy with 600 mg of Aramchol, which was monitored by MRI. It was shown that this therapy led to a decrease in the content of triglycerides in the liver, but this decrease was not statistically significant.

The ARMOR trial is a study that is still ongoing. It plans to include 150 patients with histopathology-confirmed MASH, stage 2 or 3 fibrosis, and prediabetes or type 2 diabetes. The impact of Aramchol at a dose of 300 mg twice a day or a placebo will be investigated. The primary completion date is set to be December 2024, and the study completion date is June 2027. [NCT04104321].

Cenicriviroc (CVC) is an agonist of chemokine receptors type 2 and 5. It has anti-inflammatory and antifibrotic effects. In the CENTAUR study, patients with MASH with an activity index ≥4 and fibrosis stage 2 or 3 were included [90]. They were divided into three groups: A, B, and C. Groups A and C received 150 mg of CVC or a placebo for two years, while group B received a placebo during the first year and CVC during the second. Biopsies were taken upon inclusion in the study and after the first and second year of therapy. It has been proven that the antifibrotic effect of CVC is achieved after the first year of therapy. The same was confirmed after the second year. What is significant is that patients who, after one year of treatment, had an improvement in the stage of fibrosis, also had antifibrotic effects from this drug in the following year, especially those with advanced fibrosis [90]. However, the study named AURORA, which was conducted with a similar goal, was terminated in the early stages after the analysis of the data indicated the insufficient effectiveness of the therapy [NCT03028740].

Selonsertib is an apoptosis signal-regulating kinase 1 (ASK1) inhibitor. It has a potential anti-inflammatory and antifibrotic effect on the liver. THE STELLAR studies were randomized, double-blind, placebo-controlled phase 3 studies conducted in patients with MASH and bridging fibrosis or compensated cirrhosis. The test groups received selonsertib at a dose of 6 mg or 18 mg once a day for 48 weeks, and the control group received a placebo under the same regimen. The data obtained from these studies showed that selonsertib had no antifibrotic effect in patients from these groups [91].

## 5. Conclusions

Considering the prevalence and growing number of patients with MASLD and MASH and the serious complications and consequences that occur in the advanced stages of the diseases, finding adequate pharmacotherapy is extremely important. Patients with MASLD/MASH have been proven to have an increased cardiovascular risk, and their mortality is most affected by cardiovascular diseases and its complications. Conducted studies have found potentially suitable drugs; however, additional research is necessary, in which the long-term effects, effectiveness, and the safety of these therapeutic modalities will be confirmed. Due to the complex connection between MASLD/MASH and cardiovascular diseases, the application of combined therapy is of great importance, and it is necessary to examine new possibilities.

## Figures and Tables

**Table 1:** List of drugs that have been investigated for the treatment of metabolic dysfunction-associated steatotic liver disease.

Drugs	Mechanism of Action
Obeticholic acid	Farnesoid X receptor (FXR) agonist
Tropifexor	FXR agonist
Pioglitazone	Peroxisome proliferator-activated receptor (PPAR) γ agonist
Saroglitazar	PPARα/γ agonist
Elafibranor	PPARα/δ agonist
Semaglutide	Glucagon-like peptide (GLP)-1 agonist
Liraglutide	GLP-1 agonists
Tirzepatide	GLP-1 and GIP agonist
Pegbelfermin	Fibroblast growth factors (FGF) 21 analogue
Efruxifermin	FGF21 analogue
Aldafermin	FGF19 analogue
Aramchol	Inhibitor of de novo synthesis of lipids
Cenicriviroc	Agonist of chemokine receptors 2 and 5
Selonsertib	Apoptosis signal-regulating kinase (ASK)1 inhibitor

## Data Availability

Not applicable.
